# The Coagulation Factor XIIa Inhibitor rHA-Infestin-4 Improves Outcome after Cerebral Ischemia/Reperfusion Injury in Rats

**DOI:** 10.1371/journal.pone.0146783

**Published:** 2016-01-27

**Authors:** Jennifer Krupka, Frauke May, Thomas Weimer, Ingo Pragst, Christoph Kleinschnitz, Guido Stoll, Con Panousis, Gerhard Dickneite, Marc W. Nolte

**Affiliations:** 1 CSL Behring GmbH, Marburg, Germany; 2 University of Würzburg, Department of Neurology, Würzburg, Germany; 3 CSL Limited, Bio21 Institute, Parkville, Victoria, Australia; University of Muenster, GERMANY

## Abstract

**Background and Purpose:**

Ischemic stroke provokes severe brain damage and remains a predominant disease in industrialized countries. The coagulation factor XII (FXII)-driven contact activation system plays a central, but not yet fully defined pathogenic role in stroke development. Here, we investigated the efficacy of the FXIIa inhibitor rHA-Infestin-4 in a rat model of ischemic stroke using both a prophylactic and a therapeutic approach.

**Methods:**

For prophylactic treatment, animals were treated intravenously with 100 mg/kg rHA-Infestin-4 or an equal volume of saline 15 min prior to transient middle cerebral artery occlusion (tMCAO) of 90 min. For therapeutic treatment, 100 mg/kg rHA-Infestin-4, or an equal volume of saline, was administered directly after the start of reperfusion. At 24 h after tMCAO, rats were tested for neurological deficits and blood was drawn for coagulation assays. Finally, brains were removed and analyzed for infarct area and edema formation.

**Results:**

Within prophylactic rHA-Infestin-4 treatment, infarct areas and brain edema formation were reduced accompanied by better neurological scores and survival compared to controls. Following therapeutic treatment, neurological outcome and survival were still improved although overall effects were less pronounced compared to prophylaxis.

**Conclusions:**

With regard to the central role of the FXII-driven contact activation system in ischemic stroke, inhibition of FXIIa may represent a new and promising treatment approach to prevent cerebral ischemia/reperfusion injury.

## Introduction

Ischemic stroke is a predominant disease in industrialized countries with a high rate of mortality or severe disability [1]. Despite its significance, therapeutic options in acute ischemic stroke are limited and rely mainly on recanalization strategies by pharmacological and/or mechanical thrombolysis [2, 3]. While recent studies have shown that mechanical thrombectomy leads to high recanalization rates of up to 80%, clinical outcome in these patients is highly variable, some with good results while others deteriorate despite recanalization [4–6]. It is well established that after ischemia organ function can deteriorate upon reperfusion, a process termed ischemia/reperfusion injury [7]. Respective pathophysiological mechanisms are complex and involve a myriad of distinct cellular and molecular pathways that are incompletely understood especially in the brain. Amongst these, an interplay between thrombotic and inflammatory processes seems to play a predominant role which led to the pathophysiological concept of “thrombo-inflammation” in ischemic stroke [8]. The contact activation system constitutes a framework of serially connected plasma proteins, namely coagulation factor XII (FXII), coagulation factor XI (FXI), plasma prekallikrein (PK) and high molecular weight kininogen (HK), and operates at a central position in this thrombo-inflammatory pathophysiology of ischemic stroke [9]. Amongst others, this system induces thrombus formation via the intrinsic coagulation cascade while at the same time it is linked to vascular permeability and stroke-related inflammation by the formation of short-lived kinins [10, 11]. Thus, targeted inhibition of the contact activation system appears to be a promising multifunctional approach to treat or prevent acute ischemic brain injury [12–14]. FXII as the initiator of the contact activation system becomes activated (FXIIa) when brought into contact with negatively-charged surfaces [15]. Subsequently, FXIIa cleaves FXI initiating the intrinsic pathway of coagulation and furthermore cleaves PK initiating the kallikrein-kinin system. The physiological role of FXII in coagulation processes had long been questioned since its hereditary deficiency in humans is not associated with a bleeding phenotype, which finally led to the concept that FXII is dispensable for coagulation processes *in vivo* [15–17]. This is confirmed by FXII-deficient rats and mice, which also display a normal hemostatic capacity even under surgical interventions [18, 19]. Interestingly, however, these animals are protected from experimentally-induced arterial thrombosis [18, 19] as well as from experimentally-induced ischemic stroke [20]. These observations led to the conclusion that the FXII-induced intrinsic coagulation pathway may be crucial for thrombosis but dispensable for hemostasis [17, 21, 22]. Therefore, inhibitors of FXII could present a safe therapeutic strategy in stroke [21, 23, 24]. It was previously demonstrated that the protein Infestin-4 derived from *Triatoma infestans* [25], a blood-feeding insect, recombinantly fused to human albumin (rHA; rHA-Infestin-4) is a strong FXIIa inhibitor in human, rat and mouse plasma *in vitro* [26]. Furthermore, prophylactic treatment with rHA-Infestin-4 was highly protective in a murine model of ischemic stroke without altering physiological hemostasis [26]. However, it remained to be seen whether prophylactic rHA-Infestin-4 administration is also protective in a stroke model using a different animal species and furthermore, whether rHA-Infestin-4 is still efficacious when applied in a therapeutic treatment approach during recanalization/reperfusion.

To answer these questions, we aimed in the current study to investigate the efficacy of rHA-Infestin-4 in a rat model of ischemia/reperfusion injury using both a prophylactic and a therapeutic treatment schedule. The results of this study confirm and extend previous observations and clearly indicate that FXIIa inhibition may represent a new and promising treatment approach in brain ischemia/reperfusion injury.

## Materials and Methods

### rHA-Infestin-4 expression and purification

Expression of rHA-Infestin-4 was performed as described previously [26], with the modification that the coding sequence had been recloned into expression vector pIRESneo3 and a stable Chinese hamster ovary (CHO) clone had been selected for high yield fermentation. Purification was started by concentrating the cell culture supernatant using the Centramate 500S Tangential Flow Filtration system with 30 kD membranes (Pall, Crailsheim, Germany). The concentrate was thereafter purified using Blue Sepharose 6 Fast Flow (GE Healthcare, Freiburg, Germany) and CaptureSelect Human Albumin (Life Technologies, Leiden, the Netherlands) chromatography, both according to protocols provided by the manufacturers. The eluate was finally dialysed and concentrated to around 50 mg/mL of protein.

### Transient middle cerebral artery occlusion (tMCAO) model

7–9 weeks old male CD rats (Charles River Laboratories GmbH, Sulzfeld, Germany) were subjected to tMCAO using the intraluminal filament technique [13]. Respective experiments were approved by the Regierungspraesidium Giessen (local ethics institution). Anesthesia was induced in spontaneously breathing animals in an isoflurane chamber with 5% isoflurane (CP Pharma, Burgdorf, Germany) and was subsequently maintained with 2.5% isoflurane via a face mask. During surgery, animals were placed on a heating device to ensure normothermia (37°C). After a midline skin incision in the neck, the left common carotid artery and external carotid artery were isolated and ligated. Following arteriotomy a 4.0 nylon monofilament (Ethilon^®^; Johnson & Johnson, St-Stevens-Woluwe, Belgium) with a heat-blunted tip was inserted into the left internal carotid artery and advanced cranially to the origin of the middle cerebral artery until a gentle resistance was felt. The occluding filament was left *in situ* for 90 minutes (min). Then animals were re-anesthetized and the occluding monofilament was withdrawn to allow for reperfusion.

### rHA-Infestin-4 treatment

Treatment with rHA-Infestin-4 was either done in a prophylactic or in a therapeutic setting. Within the prophylactic treatment approach, animals were intravenously (i.v.) treated with 100 mg/kg rHA-Infestin-4 15 min prior to tMCAO, whilst in the therapeutic treatment, 100 mg/kg rHA-Infestin-4 was administered i.v. directly after start of reperfusion. The dose of 100 mg/kg was chosen based on previous data which demonstrated that this dose completely protected rats from ferric chloride-induced arterial thrombosis [26]. Control animals in each study approach received an equal volume of isotonic saline (vehicle) (B. Braun, Melsungen, Germany).

### Stroke study design

Rats were randomly assigned to the treatment groups and all studies were performed in a blinded manner. Subarachnoidal hemorrhage (SAH) (as macroscopically assessed during brain sampling), death before treatment initiation and deviation from the above-mentioned study design were defined as exclusion criteria and respective animals were excluded from end-point analyses. Of the 126 rats subjected to tMCAO, 8 rats (6.4%) met at least one of the above exclusion criteria and were withdrawn from the study. Drop-out rates were evenly distributed between the groups (4 saline-treated and 4 rHA-Infestin-4-treated rats). Statistical analysis was performed on a per-protocol basis not taking into account the excluded animals.

### Determination of brain infarct area and edema formation

Within both treatment approaches (prophylactic and therapeutic treatment) brains were taken 24 hours (h) after induction of tMCAO, cut into 2-mm thick coronal sections and stained for 15 min at 37°C with 2% 2,3,5-triphenyltetrazolium chloride (TTC) (Merck Eurolab GmbH, Darmstadt, Germany) to visualize the infarct area. Stained slices were digitalized using a desk scanner and thereafter analyzed for infarct area and brain edema formation by planimetry using Image J software (National Institutes of Health, Bethesda, USA) as described previously [13]. Thereby, edema-corrected infarct areas were calculated as follows:

Infarct area(%)=100-[(AI+AC)x100/(AC x2)], whereas AI represents the total area of viable tissue of the ipsilateral (stroked) hemisphere and AC represents the total area of the contralateral (healthy) hemisphere.

The extent of brain edema was calculated according to the following equation:

Brain edema area(%)=[(AL+AI+AC)x100/(AC x2)]−100, whereas AL represents the total area of TTC-negative (ischemic) brain tissue, AI represents the total area of viable tissue of the ipsilateral (stroked) hemisphere, and AC represents the total area of the contralateral (healthy) hemisphere.

### Assessment of neurological outcome after tMCAO

Shortly before euthanasia, rats were tested for functional neurological performance. Four different evaluation systems were used: the Zea Longa Score [27], the Neuroscore [13], the RotaRod test [28–31] and the Grip Strength assay [32, 33].

The Zea Longa score ranges from 0 to 5. In detail, respective scores can be split into: The rat shows no neurological deficits (0), the rat is unable to extend affected forelimb (1), the rat circles while walking (2), the rat tumbles to its side while walking (3), the rat is unable to walk and unconsciousness is present (4), the rat is dead (5) [27].

The composite Neuroscore consists of a battery of different neurological tests in which the sum of all subitems constitutes the total neurological score (maximum score is 28, with a score of 0 indicating no neurological deficit) [13]. In detail, the test battery evaluates: postural signs with “forelimb flexion” (degree of limb flexion when animal is held by tail; 0–2 points) and “thorax twisting” (degree of body rotation when animal is held by tail; 0–2 points); gait disturbances with “circling” (straight walking = 0 points, walking towards contralateral side = 1 point, alternate circling and walking straight = 2 points, alternate circling and walking toward paretic side = 3 points, circling and/or other gait disturbance [backing, crawling, walking on digits] = 4 points, and constant circling toward paretic side = 5 points); limb placing with “forelimb placing” (normal, weak, or no placing; 0–2 points) and “hind limb placing” (normal, weak, or no placing; 0–2 points); balance on a cylindrical beam (10 cm above the floor, 3 times): 0 or 1 times falling off the beam with or without attempt to stay on the beam = 0 points, 2 times falling off the beam with or without attempt to stay on the beam = 1 to 2 points, 3 times falling off the beam with or without attempt to stay on the beam = 3 to 4 points; symmetry of muscle tone/strength with “lateral resistance” (degree of resistance against lateral push; 0–2 points) and “grasping strength” (symmetry of grasping strength onto wire cage; 0–2 points); sensory function with “grasping reflex of forepaw” (grasping onto tube when gently touched; 0–1 points) and “touching reflex” (withdrawal of forelimb when touched by needle; 0–1 points); and motility/spontaneous activity (1-minute observation): normal or slightly reduced exploratory behavior = 0 to 1 points, moving limbs without proceeding = 2 points, moving only to stimuli = 3 points, unresponsive to stimuli with normal muscle tone = 4 points, and severely decreased tone/ premortal signs = 5 points.

The RotaRod test [28–31] is a functional performance test in which animals are subjected to a rotating rod with forced motor activity being applied. Amongst others, this test evaluates balance and motor coordination of the animals. At the test day, i.e. 24 h after induction of tMCAO, animals were set on the RotaRod cylinder (TSE Systems, Bad Homburg, Germany), whose speed was increased successively: 0–10 rounds per min (rpm) within 5 seconds (sec); 10–30 rpm within 55 sec; 30 rpm for 30 sec; 30–40 rpm within 10 sec, 40 rpm for 20 sec. Here, the maximum duration on the rotating cylinder of each rat was measured. The test stopped immediately when the animal fell off the cylinder. In order to adapt the animals to the RotaRod test, the animals were trained twice 7 days before tMCAO using the same test procedure as described above. Thereafter, baseline values for each animal were determined 6 days before tMCAO within one RotaRod measurement and were set to 100%. RotaRod data are shown in percent compared to baseline.

Functional performance was also investigated by determining the gripping strength using the Grip Strength meter (TSE Systems, Bad Homburg, Germany) 24 h after induction of tMCAO. Also here, animals were trained twice (with 5 grip strength measurements each) 7 days before tMCAO. Baseline values of each rat were evaluated 6 days before tMCAO with 5 grip strength measurements in total. Here, the mean of the three best values was calculated and was set to 100%. Grip strength data are presented as percentage compared to individual baseline values.

### Determination of aPTT, PT and FXIIa activity in plasma samples

Within the prophylactic approach blood samples were taken 24 h after MCAO for the analysis of activated partial thromboplastin time (aPTT; assay to evaluate the FXII-driven intrinsic pathway of coagulation), prothrombin time (PT; assay to evaluate the extrinsic pathway of coagulation) and FXIIa activity. Those tests were performed *in vitro* in plasma samples according to the manufacturer’s protocol using the BCS^®^ XP system (Siemens Healthcare Diagnostics Products GmbH, Marburg, Germany).

### Statistical evaluation

All data are shown as mean ± standard error of the mean (SEM). Statistical analyses were performed using the GraphPad Prism 5.0 software (GraphPad Software, Inc., La Jolla, CA, USA). Infarct area, brain edema formation as well as all neurological assays were analyzed mortality-adjusted (as defined in study protocol; dead animals were given the worst value) using the Mann-Whitney U-test. Mortality was analyzed using the Chi-square test. FXIIa activity, aPTT and PT were analyzed using the unpaired t-test. A p-value below 0.05 was considered statistically significant.

## Results

### Prophylactic application of rHA-Infestin-4 significantly improves outcome after tMCAO in rats

**Fig 1:**
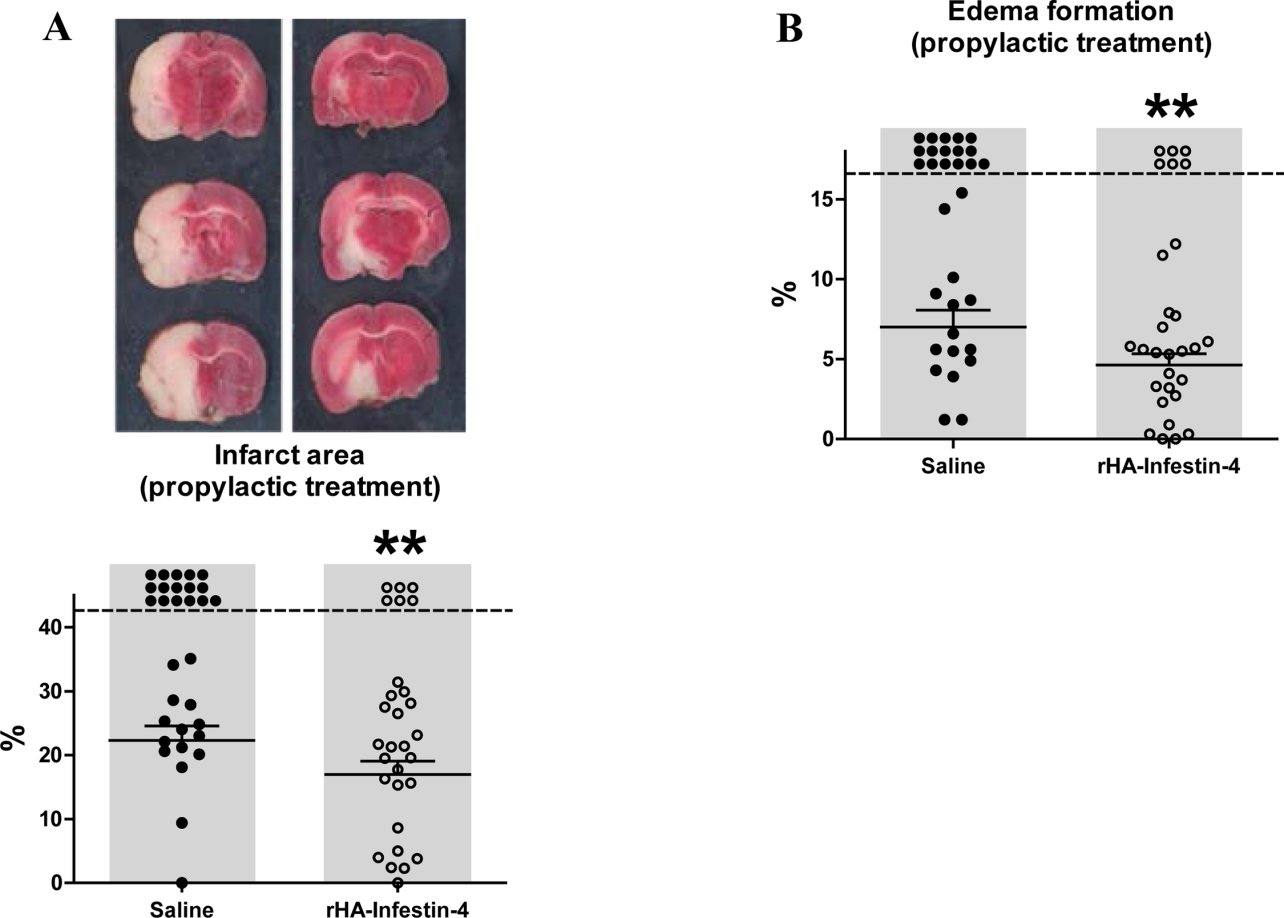
Prophylactic treatment with rHA-Infestin-4 reduces infarct area and brain edema formation after tMCAO in rats. **(A)** Upper panel shows representative 2,3,5-triphenyltetrazolium chloride stainings of 3 coronal rat brain sections 24 h after transient middle cerebral artery occlusion (tMCAO) in rats. Rats were treated with 100 mg/kg rHA-Infestin-4 or saline (control) 15 min prior to tMCAO. tMCAO was held up for 90 min followed by reperfusion until 24 h. Corresponding individual infarct areas, analyzed by planimetry, are shown in the lower panel. Prophylactic rHA-Infestin-4 treatment significantly reduced infarct areas compared to saline treated rats (n = 29-31/group). **p<0.01, Mann-Whitney test compared to saline treated animals. Dots above the dashed horizontal line indicate dead animals per group. Mortality was included in statistical analysis. Horizontal solid lines indicate mean values (+SEM) of surviving animals. **(B)** In addition, brain edema formation 24 h after tMCAO was reduced in rHA-Infestin-4 treated rats compared to saline treated control rats (n = 29-31/group). **p<0.01, Mann-Whitney test compared to saline treated animals. Dots above the dashed horizontal line indicate dead animals per group. Mortality was included in statistical analysis. Horizontal solid lines indicate mean values (+SEM) of surviving animals.

**Fig 2:**
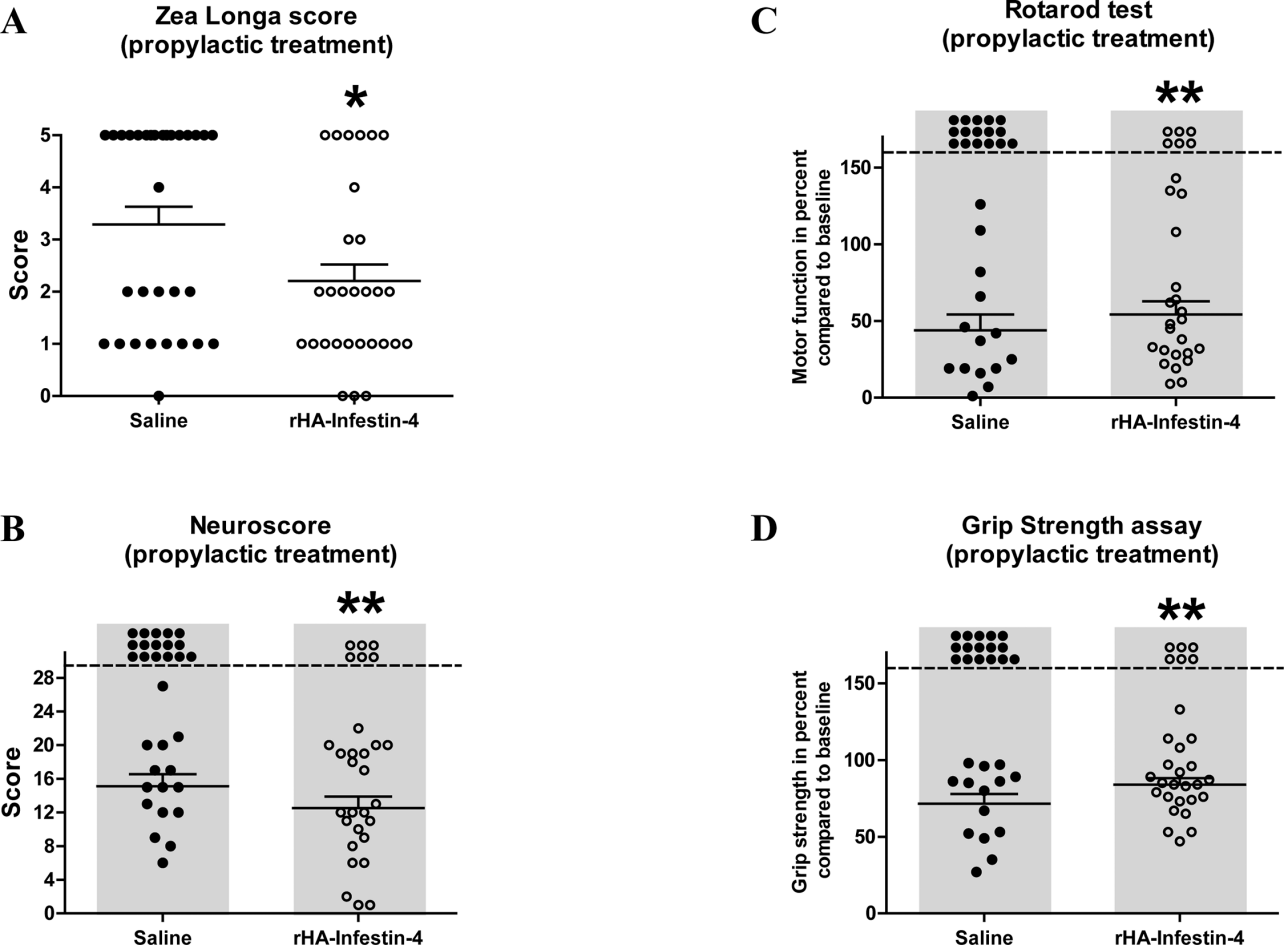
Prophylactic treatment with rHA-Infestin-4 ameliorates neurological outcome after tMCAO in rats. **(A)** Zea Longa score 24 h after transient middle cerebral artery occlusion (tMCAO) in rats. Rats were treated with 100 mg/kg rHA-Infestin-4 or saline (control) 15 min prior to tMCAO. tMCAO was held up for 90 min followed by reperfusion until 24 h. Prophylactic rHA-Infestin-4 treatment significantly reduced the Zea Longa score compared to saline treated rats (n = 29-31/group). *p<0.05, Mann-Whitney test compared to saline treated animals. Horizontal solid lines indicate mean values (+SEM). **(B)** In addition, the Neuroscore was reduced in rHA-Infestin-4 treated rats compared to saline treated rats 24 h after tMCAO (n = 29-31/group). **p<0.01, Mann-Whitney test compared to saline treated animals. Dots above the dashed horizontal line indicate dead animals per group. Mortality was included in statistical analysis. Horizontal solid lines indicate mean values (+SEM) of surviving animals. **(C)** Functional performance on RotaRod and **(D)** grip strength in percent compared to the individual baseline of saline treated and rHA-Infestin-4 treated animals 24 h after tMCAO. rHA-Infestin-4 significantly improved functional performance in these two assays compared to saline treated controls (n = 28-30/ group). **p<0.01 between rHA-Infestin-4 treated and saline treated animals, Mann-Whitney test. Dots above the dashed horizontal line indicate dead animals per group. Mortality was included in statistical analysis. Horizontal solid lines indicate mean values (+SEM) of surviving animals.

To assess the efficacy of rHA-Infestin-4 in acute focal cerebral ischemia in rats, we chose the tMCAO thread model [13], which to some extent mimics the clinical situation of rapid recanalization after mechanical thrombectomy. First, a prophylactic treatment approach was applied in which rats were given rHA-Infestin-4 prior to tMCAO. At 24 hours after induction of tMCAO, infarct areas of rHA-Infestin-4-treated rats were significantly reduced compared to saline-treated controls (22.3±2.3% [controls] vs. 17.0±2.1% [100 mg/kg rHA-Infestin-4]; p<0.01 (mortality-adjusted); Fig 1A). We furthermore analyzed effects on brain edema formation, a space-occupying process amongst others evolving during inflammatory pathomechanisms of acute ischemic stroke in rodents as well as stroke patients [13, 34]. In line with the role of FXII as initiator of the contact activation system [35], brain edema formation was significantly decreased in rats treated with the FXIIa inhibitor rHA-Infestin-4 compared to control rats (7.0±1.1% [control] vs 4.6±0.7 [100 mg/kg rHA-Infestin-4]; p<0.01 (mortality-adjusted); Fig 1B). Importantly, the reduced infarct area and brain edema formation were functionally relevant, since neurological functions also improved after rHA-Infestin-4 treatment (Fig 2). Compared to controls, rHA-Infestin-4 treated rats demonstrated a reduced Zea Longa score as well as a reduced Neuroscore 24 h after induction of tMCAO (Zea Longa score: 3.3±0.3 [control] vs 2.2±0.3 [100 mg/kg rHA-Infestin-4], p<0.05, Fig 2A; Neuroscore: 15.1±1.4 [control] vs 12.5±1.4 [100 mg/kg rHA-Infestin-4], p<0.01 (mortality-adjusted), Fig 2B). In addition, rHA-Infestin-4 treatment also improved the functional performance in the more complex RotaRod test as well as the individual grip strength assay 24 h after induction of tMCAO compared to saline treated controls (RotaRod: 43.9±10.3% [control] vs 54.2±8.6% [100 mg/kg rHA-Infestin-4], p<0.01 (mortality-adjusted), Fig 2C; grip strength: 71.4±6.4% [control] vs 83.9±4.3% [100 mg/kg rHA-Infestin-5], p<0.01 (mortality-adjusted), Fig 2D). Mortality is included within the statistical evaluation of aforementioned analyses. However, when analyzing mortality in isolation, prophylactic rHA-Infestin-4 treatment remarkably improved survival compared to saline treated controls (mortality: 52% [control] vs 21% [100 mg/kg rHA-Infestin-4], p<0.05, Chi-square test).

In addition to the functional and histopathological evaluations, terminal blood samples were drawn and aPTT, PT and FXIIa activity analyzed (Table 1). Data clearly demonstrate that FXIIa inhibition via rHA-Infestin-4 persisted until 24 h after application. Compared to controls, FXIIa activity was significantly decreased (191±9.8% of the norm [control] vs 142±9.3% of the norm [100 mg/kg rHA-Infestin-4], p<0.01, Table 1), the corresponding aPTT prolonged (16.8±0.71 sec [control] vs 41.7±3.86 sec [100 mg/kg rHA-Infestin-4], p<0.001, Table 1), whilst the PT was unchanged (8.47±0.12 sec [control] vs 8.54±0.25 sec [100 mg/kg rHA-Infestin-4], p>0.05, Table 1).

**Table 1:** Prophylactic rHA-Infestin-4 application is still active 24 h after tMCAO in rats.

No.	Treatment	aPTT (sec)	PT (sec)	FXIIa activity (% of the norm)
**1**	Isotonic saline	16.8 ± 0.71	8.47 ± 0.12	191 ± 9.8
**2**	rHA-Infestin-4, 100 mg/kg	41.7 ± 3.86***	8.54 ± 0.25	142 ± 9.3**

Activated partial thromoplastin time (aPTT), prothrombin time (PT) and FXIIa activity values 24 h after transient middle cerebral artery occlusion (tMCAO) in rats. Rats were treated with 100 mg/kg rHA-Infestin-4 or saline (control) 15 min prior to tMCAO. tMCAO was held up for 90 min followed by reperfusion until 24 h.

N = 11-12/group; mean±SEM

**p<0.01

***p<0.0001, unpaired t-test.

### Therapeutic application of rHA-Infestin-4 significantly improves outcome after tMCAO in rats

**Fig 3:**
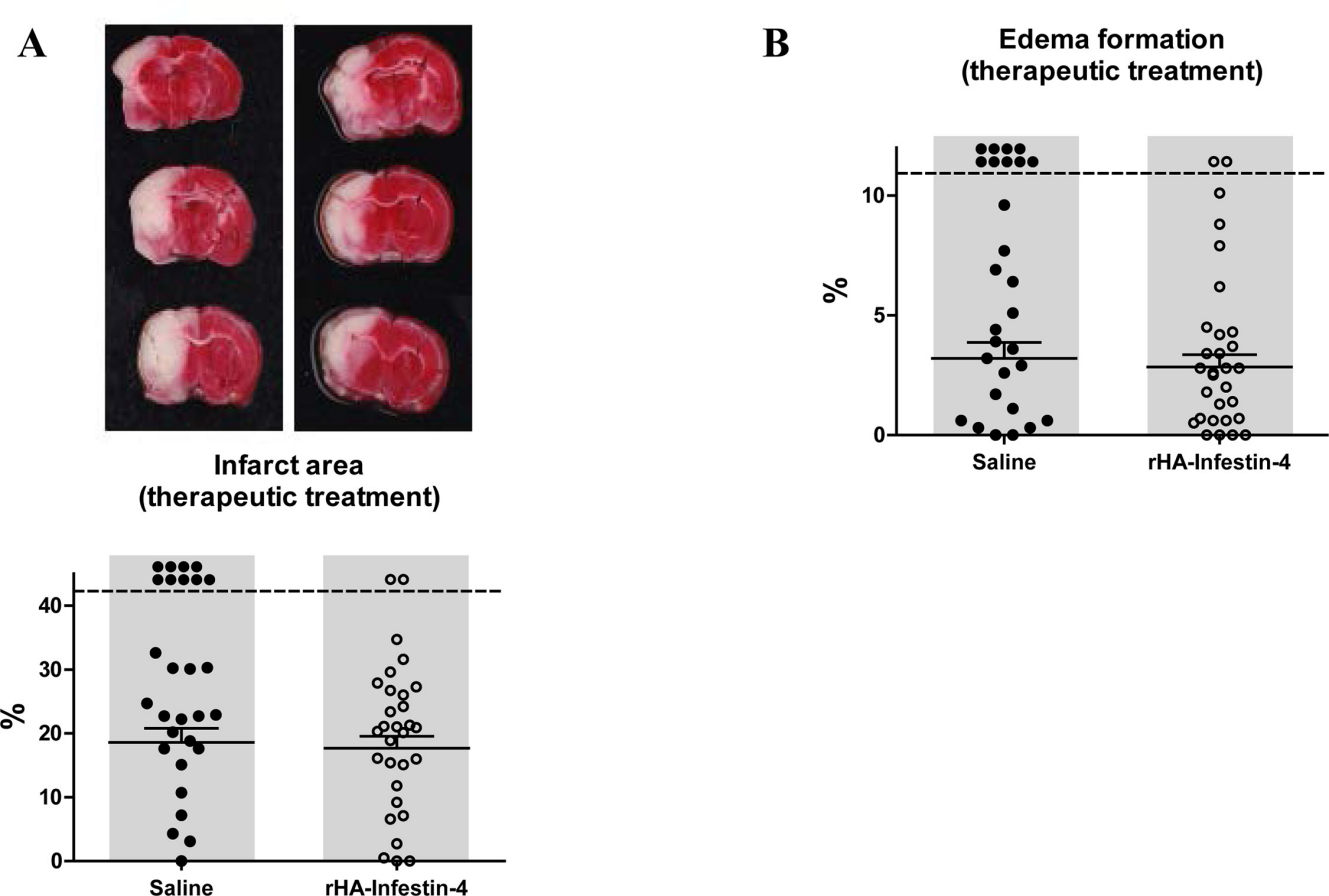
Therapeutic treatment with rHA-Infestin-4 tends to reduce infarct area and brain edema formation after tMCAO in rats. **(A)** Upper panel shows representative 2,3,5-triphenyltetrazolium chloride stainings of 3 coronal rat brain sections 24 h after transient middle cerebral artery occlusion (tMCAO) in rats. Rats were subjected to 90 min of tMCAO followed by reperfusion until 24 h. Rats were treated with 100 mg/kg rHA-Infestin-4 or saline (control) directly after start of reperfusion. Corresponding individual infarct areas, analyzed by planimetry, are shown in the lower panel. A trend towards reduced infarct areas could be detected after therapeutic rHA-Infestin-4 treatment compared to saline treated rats (n = 28-30/group). p<0.1 and >0.05, Mann-Whitney test compared to saline treated animals. Dots above the dashed horizontal line indicate dead animals per group. Mortality was included in statistical analysis. Horizontal solid lines indicate mean values (+SEM) of surviving animals. **(B)** In addition, a trend towards reduced brain edema formation 24 h after tMCAO was visible in rHA-Infestin-4 treated rats compared to saline treated control rats (n = 28-30/group). p<0.1 and >0.05, Mann-Whitney test compared to saline treated animals. Dots above the dashed horizontal line indicate dead animals per group. Mortality was included in statistical analysis. Horizontal solid lines indicate mean values (+SEM) of surviving animals.

**Fig 4:**
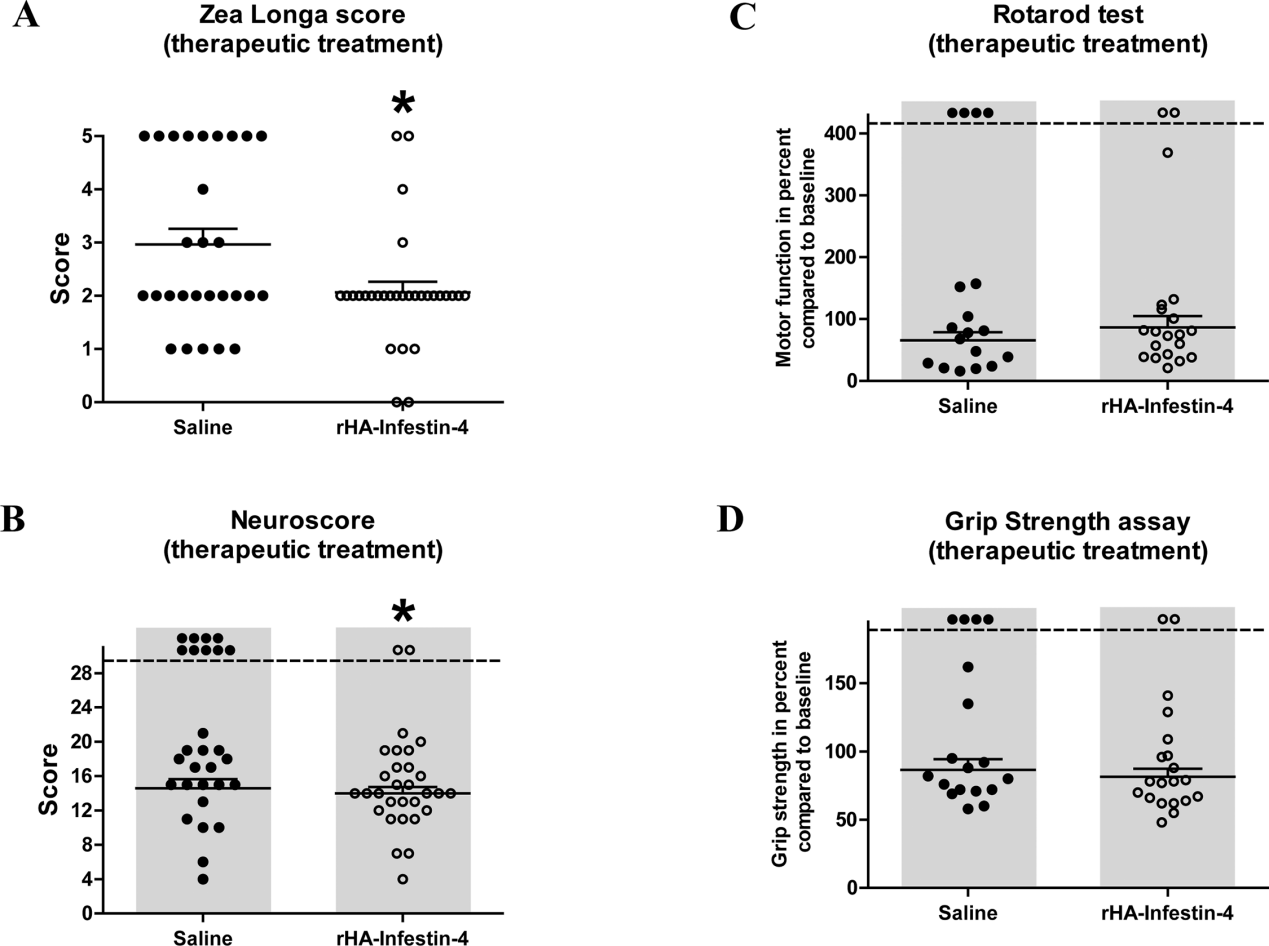
Therapeutic treatment with rHA-Infestin-4 improves neurological performance after tMCAO in rats. **(A)** Zea Longa score 24 h after transient middle cerebral artery occlusion (tMCAO) in rats. Rats were subjected to 90 min of tMCAO followed by reperfusion until 24 h. Rats were treated with 100 mg/kg rHA-Infestin-4 or saline (control) directly after start of reperfusion. Therapeutic rHA-Infestin-4 treatment significantly reduced the Zea Longa score compared to saline treated rats (n = 28-30/group). *p<0.05, Mann-Whitney test compared to saline treated animals. Horizontal solid lines indicate mean values (+SEM). **(B)** In addition, the Neuroscore was reduced in rHA-Infestin-4 treated rats compared to saline treated rats 24 h after tMCAO (n = 28-30/group). *p<0.05, Mann-Whitney test compared to saline treated animals. Dots above the dashed horizontal line indicate dead animals per group. Mortality was included in statistical analysis. Horizontal solid lines indicate mean values (+SEM) of surviving animals. **(C)** Functional performance on RotaRod and **(D)** grip strength in percent compared to the individual baseline of saline treated and rHA-Infestin-4 treated animals 24 h after tMCAO. rHA-Infestin-4 had no effect in these two assays compared to saline treated controls (n = 18-20/ group). p>0.05 between rHA-Infestin-4 treated and saline treated animals, Mann-Whitney test. Dots above the dashed horizontal line indicate dead animals per group. Mortality was included in statistical analysis. Horizontal solid lines indicate mean values (+SEM) of surviving animals.

To evaluate if rHA-Infestin-4 treatment is also effective when applied after stroke onset, e.g. immediately during reperfusion, a 2^nd^ study was initiated in which rHA-Infestin-4 was applied directly after 90 min of tMCAO, when the vessel occluding thread was withdrawn. As before, functional and histopathological evaluations were performed 24 h after induction of tMCAO. Interestingly, therapeutic application of rHA-Infestin-4 had no significant effect on infarct area assessed by TTC staining 24 h after induction of tMCAO, albeit, compared to controls, a trend towards a decreased infarct area was visible after rHA-Infestin-4 treatment (18.6±2.2% [control] vs 17.7±1.9% [100 mg/kg rHA-Infestin-4], p<0.1 and >0.05 (mortality-adjusted); Fig 3A). Comparable results were also observed for brain edema formation, i.e. a trend towards reduced brain edema formation after rHA-Infestin-4 treatment (3.2±0.7% [control] vs 2.8±0.5% [100 mg/kg rHA-Infestin-4], p<0.1 and >0.05 (mortality-adjusted); Fig 3B). Notably, neurological functions were still improved after therapeutic rHA-Infestin-4 treatment (Fig 4). The Zea Longa score as well as the Neuroscore were ameliorated by rHA-Infestin-4 in comparison to saline controls (Zea Longa score: 3.0±0.3 [control] vs 2.1±0.2 [100 mg/kg rHA-Infestin-4], p<0.05, Fig 4A; Neuroscore: 14.6±1.1 [control] vs 14±0.7 [100 mg/kg rHA-Infestin-4], p<0.05 (mortality-adjusted), Fig 4B). Remarkably, also after therapeutic rHA-Infestin-4 treatment, mortality was significantly reduced compared to saline treated controls (32% [control] vs 7% [100 mg/kg rHA-Infestin-4], p<0.05, Chi-square test). Within the RotaRod test and grip strength assay, no significant effect was detectable after therapeutic rHA-Infestin-4 treatment compared to controls (RotaRod: 65.9±12.6% [control] vs 86.6±18.3% [100 mg/kg rHA-Infestin-4], p>0.05 (mortality-adjusted), Fig 4C; grip strength: 86.6±7.7% [control] vs 81.4±5.9% [100 mg/kg rHA-Infestin-5], p>0.05 (mortality-adjusted); Fig 4D).

## Discussion

Here we show that the FXIIa inhibitor rHA-Infestin-4 improves outcome after cerebral ischemia/reperfusion injury in rats in two clinically meaningful scenarios, i.e. a prophylactic treatment approach for patients at risk of developing an ischemic stroke and a therapeutic treatment approach during recanalization/reperfusion. Following prophylactic rHA-Infestin-4 treatment, infarct area and brain edema formation were reduced whilst neurological capacities and survival were improved. In comparison, using a therapeutic treatment approach, whilst neurological outcome and survival were still significantly improved, the overall effects were less pronounced compared to prophylaxis.

With this study we demonstrate for the first time that FXIIa inhibition via rHA-Infestin-4 protects from ischemic stroke i) in rats, thus excluding that neuroprotective effects of FXIIa inhibition via rHA-Infestin-4 could only be observed in mice [26], a commonly used species for preclinical studies on ischemic stroke [36] and ii) within a clinically-relevant therapeutic treatment regime, i.e. when applied after ischemic onset at initiation of recanalization/reperfusion. The enhanced efficacy for prophylactic administration is consistent with literature data demonstrating that the contact activation system is already activated during ischemic stroke [37–39] and suggests that clinical applicability of FXIIa inhibition may be more effective in prophylactic scenarios. Respective scenarios include prevention of thromboembolic complications in patients undergoing vascular procedures/ surgeries [40, 41] (e.g. angiography, carotid endarterectomy or coronary bypass surgery) or clinical settings where blood becomes exposed to reactive artificial biomaterials [42–46] (e.g. cardiac surgery using a cardiopulmonary bypass, extracorporeal membrane oxygenation, hemodialysis or the use of endoprothetics), but also in patients with atrial fibrillations or other pre-existing conditions [47].

However, since new clinical data from mechanical recanalization studies clearly indicate that successful recanalization cannot be directly equated with functional improvement [4], data from our therapeutic study arm are likewise promising and could lead to new treatment options to combat brain ischemia/reperfusion injury following mechanical recanalization of occluded vessels.

Nevertheless, our data strongly support the view that the contact activation system is crucially involved in brain ischemia/reperfusion injury [38, 39, 48] and are in line with other studies evaluating the role of further constituents of the contact activation system in ischemic stroke [13, 14, 49, 50]. For example, Goeb and colleagues demonstrated that genetic depletion of plasma kallikrein as well as targeted inhibition of plasma kallikrein via an antibody reduced infarct volume and improved neurological parameters following murine tMCAO [14], whilst antibody-mediated inhibition of FXI [50] as well as kininogen deficiency [49] likewise improved outcome following ischemic stroke in mice. Furthermore, when mice or rats were subjected to tMCAO, administration of C1-esterase inhibitor (C1-INH), the physiological inhibitor of the contact activation system [51], also reduced brain infarction and improved neurological behavior [13], confirming the relevance of the contact activation system in stroke.

The beneficial effects of rHA-Infestin-4 in tMCAO observed in our study are most likely due to the antithrombotic and anti-inflammatory properties of FXIIa inhibition [20, 44, 52, 53]. Anti-inflammatory effects of rHA-Infestin-4 application could be demonstrated in this study by reduction of edema formation in infarcted brain tissue as well as in another study applying rHA-Infestin-4 in a murine traumatic brain injury model [54], whereas antithrombotic effects of rHA-Infestin-4 have been reported in several thrombosis models [26]. Of note, also C1-INH treatment in rodent tMCAO exerted its beneficial effects via antithrombotic and anti-inflammatory mechanisms [13].

In addition to the beneficial effects of FXIIa inhibition via rHA-Infestin-4 in murine [26] and rat (data from the current study) models of tMCAO, which are in line with data from FXII-deficient mice in tMCAO [20], rHA-Infestin-4 has also been applied in models of atherothrombosis, itself being a trigger for stroke [55]. In this study, utilizing a murine *in vivo* as well as a human *ex vivo* atherothrombosis model, rHA-Infestin-4 was shown to be effective in reducing thrombus size and interfering with thrombus stability. Furthermore, rHA-Infestin-4 largely prevented ischemic brain lesion development in two murine models of silent brain ischemia employing fibrin clots or microbeads for ischemia induction [41]. Overall, rHA-Infestin-4 has repeatedly demonstrated to be effective in several animal models of ischemic stroke or associated pathologies.

Current therapeutic options for prevention of lesion enlargement and recurrent thromboembolism with anticoagulants and platelet aggregation inhibitors are still associated with a risk of intracranial hemorrhages [56–59]. Therefore not only clinically effective but also safer therapeutic options are warranted. Based on a new concept, FXII seems to be relevant for thrombosis but not for physiological coagulation processes *in vivo* [15–17]; making FXII the ideal target for a safe treatment approach in stroke. In line with multiple other reports on FXII-deficient humans [60, 61] and FXII-deficient [18, 19] or -inhibited animals [17], we did not find any evidence for an increased bleeding tendency of rHA-Infestin-4 treatment in this study and recent studies in murine models of silent brain ischemia [41], further emphasizing the opportunity for a safe treatment approach using FXIIa inhibitors.

Some limitations to the use of rHA-Infestin-4 in clinical scenarios exist. First, since Infestin-4, the active component of rHA-Infestin-4, is an insect-derived protein [25], a potential immunogenicity issue in human use cannot be excluded. Second, although fusion of Infestin-4 to human albumin dramatically improved the half-life of this protein in mice, i.e. from 0.3 to 4.6 hours [26], pharmacokinetic characteristics of this molecule may not be optimal for prophylactic regimes in stroke prevention. To overcome these limitations, a specific, fully human monoclonal antibody against FXIIa has recently been generated [44]. Furthermore, a limitation of this study is that effects of rHA-Infestin-4 treatment were only investigated until 24 hours after onset of ischemia. Therefore, future studies should also evaluate long term outcome of treatment. However, it is reassuring that C1-INH treatment in murine tMCAO already demonstrated longer lasting beneficial effects on stroke outcome [13].

If the beneficial preclinical results of FXIIa inhibition, or inhibition of other components of the contact activation system, translate into a safe and effective treatment for stroke patients remains to be seen. However, a study demonstrating that the incidence of ischemic stroke is reduced in FXI-deficient patients [62] provides support for this premise.

In summary, we have demonstrated that FXIIa inhibition via rHA-Infestin-4 improves stroke outcome in clinically-meaningful scenarios. With regard to the central role of the FXII-driven contact activation system in stroke, inhibition of FXIIa may represent a new and promising treatment approach for brain ischemia/reperfusion injury.
